# Cellular Heterogeneity During Arterial Aging

**DOI:** 10.1111/acel.70597

**Published:** 2026-06-23

**Authors:** He Xu, Paul‐Lennard Mendez, Dimitri Kasakovski, Judith Sluimer

**Affiliations:** ^1^ Department of Pathology Cardiovascular Research Institute Maastricht (CARIM), Maastricht University Maastricht the Netherlands; ^2^ Berlin Institute of Health at Charité–Universitätsmedizin Berlin Berlin Germany; ^3^ Max‐Delbrück‐Center for Molecular Medicine in the Helmholtz Association (MDC), Berlin Institute for Medical Systems Biology (BIMSB) Berlin Germany; ^4^ Department for Renal and Hypertensive Rheumatological and Immunological Diseases (Medical Clinic II), RWTH Aachen Aachen Germany; ^5^ British Heart Foundation Centre for Cardiovascular Science, Queen's Medical Research Institute The University of Edinburgh Edinburgh UK

**Keywords:** arterial aging, cell–cell communication, cellular senescence, extracellular matrix remodeling, inflammaging, single‐cell RNA sequencing, vascular heterogeneity

## Abstract

Arterial aging is a major risk factor for cardiovascular disease and is associated with progressive changes in vascular structure and function, including arterial stiffening, reduced elasticity, extracellular matrix remodeling, chronic low‐grade inflammation, and accumulation of senescence‐associated cell states. Recent advances in single‐cell RNA sequencing (scRNA‐seq) have provided new opportunities to resolve the cellular heterogeneity underlying these age‐related alterations in the arterial wall. In this review, we summarize current single‐cell studies of arterial aging by focusing first on key phenotypic programs, including cellular senescence, extracellular matrix remodeling, inflammaging, and altered intercellular communication, and then discuss how these programs are reflected in endothelial cells, smooth muscle cells, fibroblasts, and immune cells. Across studies, aging is recurrently associated with endothelial dysfunction, smooth muscle cell phenotypic modulation, fibroblast‐related matrix remodeling, and immune activation, although the degree of conservation varies depending on species, vascular bed, sex, and disease context. We further discuss emerging evidence that vascular aging involves not only cell‐intrinsic transcriptional changes but also alterations in communication networks across the arterial wall. Although current single‐cell studies have substantially improved our understanding of arterial aging, important limitations remain, including inconsistent cell‐state annotation across studies, incomplete functional validation, and limited spatial and epigenetic resolution. Future integration of cross‐species analyses with spatial transcriptomics, single‐cell epigenomic approaches, and functional studies will help refine the cellular framework of arterial aging and improve its translational relevance.

## Graphical abstract

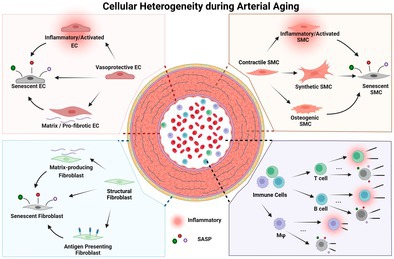

## 1 Introduction

Global aging is accelerating at an unprecedented rate. According to the World Health Organization (WHO 2021), the proportion of the global population aged 65 years and older will reach 22% by 2050. Aging is characterized by a wide array of biological processes, and researchers have identified key hallmarks that drive this complex phenomenon, including: cellular senescence, extracellular matrix (ECM) alterations, chronic inflammation, and altered intercellular communication (Kroemer et al. 2025; López‐Otín et al. 2023). As aging progresses, cardiovascular diseases (CVDs) have emerged as one of the most common health threats among the elderly population. Arteries play a crucial role in maintaining overall health by transporting circulating cells, oxygen, and nutrients to various tissues and organs (Najjar et al. 2005). However, as the aging process advances, the structure and function of arteries undergo significant changes. These changes are mainly characterized by increased arterial stiffness and reduced elasticity, resulting in hypertension or other severe cardiovascular conditions, such as atherosclerosis and aneurysms (Lee and Oh 2010; Weinsaft and Edelberg 2001; Zha et al. 2022). Arterial aging also impacts smaller arteries and the microvascular system, resulting in widespread cardiovascular dysfunction (Saz‐Lara et al. 2023). Therefore, these age‐dependent changes make aging a major risk factor for CVDs.

In the arterial system, four key cell types—endothelial cells (ECs), vascular smooth muscle cells (SMCs), fibroblasts, and immune cells—play vital roles in maintaining the structural integrity and function of the arterial wall. In vascular inflammation, the infiltrates consist primarily of T lymphocytes, monocytes, macrophages, and dendritic cells (DCs), which interact with resident vascular wall cells, including SMCs, ECs, and fibroblasts (Terekhova et al. 2023). During aging, arterial cells undergo significant structural and functional changes, leading to overall declines in arterial health. These changes include a shift in cellular phenotypes, increased inflammation, and impaired tissue integrity (Kitada et al. 2016; Shehadeh et al. 2011; Tinajero and Gotlieb 2020; van der Linden et al. 2024).

Recent advances in single‐cell transcriptomics have provided unprecedented resolution into the heterogeneity of cells within tissues, offering deeper insights into the molecular mechanisms driving arterial aging (Cheng et al. 2024). These technologies have revealed diverse transcriptional states across vascular cell populations during aging. This review aims to synthesize major single‐cell studies into key cell phenotypes that characterize arterial aging and to integrate these findings across vascular cell types and different species. Through this, we gain insights into the unique functions of distinct cell subpopulations during arterial aging.

## 2 Key Phenotypic Programs Driving Arterial Aging

In the following sections, we will summarize important key hallmarks of vascular aging and integrate phenotypic changes across cell types based on findings from single‐cell studies. Importantly, while many phenotypic alterations such as senescence and inflammation are shared across vascular cell types, their functional consequences are highly cell type‐specific. Therefore, we first outline key aging‐associated programs and subsequently discuss how these manifest within individual vascular cell populations.

### 2.1 Cellular Senescence

Cellular senescence is a well‐established hallmark of vascular aging and has been extensively reviewed in the vascular context (Bloom et al. 2023; Kovacic et al. 2011). In brief, senescence is a protective response to acute or chronic damage, where cells permanently lose the ability to divide and acquire a proinflammatory secretory phenotype (SASP). As humans age, senescent cells accumulate driven by factors such as DNA damage, oxidative stress, and telomere shortening (Gorgoulis et al. 2019).

With the possibility of analyzing transcriptomes at single‐cell resolution, senescence can now be resolved as a heterogeneous and cell type‐specific transcriptional state rather than a uniform phenotype. For instance, single‐cell analyses support that senescence represents a transcriptomic continuum rather than a binary state. Mapping of single‐cell transcriptomic data from senescent ECs on a pseudotime trajectory revealed gradual transitions from proliferative to senescent states with distinct intermediate transcriptional programs (Ahn et al. 2025; Tao et al. 2024). Complementing this, recent machine learning‐based frameworks such as SenCID use bulk or single‐cell transcriptomic data to enable the identification of multiple senescence‐associated identities across cell types and conditions, further highlighting the diversity of senescent phenotypes and their context dependence.

Single‐cell and spatial transcriptomic analyses of PCSK9‐induced atherosclerosis have identified multiple senescence‐enriched populations across SMCs, fibroblasts, and immune cells, defined by composite gene signatures (e.g., SenMayo, CellAge) rather than canonical markers alone (Avelar et al. 2020; Mazan‐Mamczarz et al. 2025; Saul et al. 2022). These data indicate that classical markers such as p16 or p21 incompletely capture senescent states in vivo and that senescence is embedded within broader functional programs, including ECM remodeling and inflammation. Importantly, by integrating the most consistently enriched markers across senescent clusters, the authors derived a vascular senescence scoring system that was not only applicable within the original atherosclerosis model but also transferable to independent contexts, including doxorubicin‐induced senescence in mice and human senescent SMCs, as well as human plaque SMC subtypes (Mazan‐Mamczarz et al. 2025). Of note, many of the consistently enriched senescence‐associated transcripts encode secreted or cell surface proteins, rendering them of interest as potential senescence biomarkers or therapeutic targets for new senolytic agents.

Taken together, single‐cell studies have redefined vascular senescence as a dynamic and continuous process and enabled new tools to identify the heterogenous population of senescent cells in age‐related vascular diseases across species.

### 2.2 Extracellular Matrix Remodeling

ECM remodeling is a central feature of arterial aging and a major determinant of arterial stiffness. At the cellular level, this process reflects changes across ECs, SMCs, and fibroblasts that result in arterial stiffening (Chan and Fiscus 2004; Hoffmann et al. 1998; Kielty et al. 2007; Krajnik et al. 2023; Lacolley et al. 2017; Osherov et al. 2011; Reed et al. 2022; Stenmark et al. 2013; Verhamme and Hoylaerts 2006). These changes in biochemical properties disrupt cellular homeostasis and are major contributors to chronic inflammation (Lai et al. 2026).

Single‐cell transcriptomic studies have refined the view on aging‐related ECM changes by demonstrating that remodeling arises from distinct and dynamic cellular states rather than uniform increases in matrix production. Notably, our own work combining murine and human single‐cell transcriptomics revealed functionally distinct fibroblast subsets, including CD55^+^, CXCL14^+^, and LOX^+^ populations, which differentially contribute to collagen accumulation or collagen crosslinking and arterial stiffening (van Kuijk et al. 2023). SMCs adopt synthetic, secretory states present in young and aged mice while there appears to be a decline in expression of key ECM transcripts (e.g., *Eln*, *Dcn*, encoding for Elastin and Decorin) and loss of a specific, proliferative SMC subset with age (Rivera et al. 2024). This is accompanied by a maladaptive mechano‐response, driven by upregulation of the mechano‐sensitive ion channel Piezo1 (Rivera et al. 2024). A recent meta study combined single cell RNAseq data from human ECs of various vascular beds, highlighting a shift in ECM‐ and glycosylation‐associated transcripts across EC subtypes. Similar to SMCs, ECs from aged humans display alterations in mechano‐signaling that accompany changes in the ECM (Dobner et al. 2025).

Beyond a link of ECM to cell state, integrative single‐cell analyses highlight that ECM remodeling is tightly linked to altered intercellular communication. Tools developed for differential intercellular communication analysis of aging atlases such as scAgeCom (Lagger et al. 2021) and scDiffCom (Lagger et al. 2023) revealed reduced collagen–integrin signaling and downregulation of angiogenesis‐ and ECM‐related gene programs. These alterations likely reflect a functional disruption of ECM‐mediated signaling despite increased ECM deposition, highlighting a shift from structural remodeling toward signaling imbalance in aging arteries.

Although studies resolving transcriptomic profiles of the aging vasculature spatially are yet missing, spatial transcriptomics of murine atherosclerotic plaques, which include senescent cells, reveal a *HMOX1*+ and *TREM2*+ macrophage subset expressing ECM regulators that could contribute to fibrosis in the shoulder region of the plaque (Pauli et al. 2025).

### 2.3 Chronic Inflammation

Chronic low‐grade inflammation, termed inflammaging, is a hallmark of aging characterized by persistent activation of immune pathways in the absence of acute infection. It is associated with increased circulating cytokines, impaired immune regulation, and contributes to the development of cardiovascular diseases (Ajoolabady et al. 2023; Aranda et al. 2025).

Single‐cell transcriptomic studies have refined the concept of inflammaging by showing that inflammatory signaling is not uniformly increased across all cells but instead arises from specific cell types and cellular states. Across tissues, these analyses consistently identify subsets of immune cells—particularly macrophages—as major contributors to age‐associated inflammatory signatures, alongside an increasing fraction of cells exhibiting senescence‐associated transcriptional programs (Majewska and Krizhanovsky 2025; Wells et al. 2025). In parallel, aging is associated with increased transcriptional variability, or noise, between cells, indicating that inflammaging reflects a shift in cell state distribution and increased heterogeneity rather than a coordinated global upregulation of inflammatory pathways (Elyahu et al. 2019; Grover et al. 2016; Martinez‐Jimenez et al. 2017). Importantly, single‐cell data allows to distinguish between a general increase in proportion of cells expressing inflammatory markers and elevated expression levels within cells, a differentiation that is not possible using bulk analyses. Single cell transcriptional profiling supports a model in which relatively small subpopulations disproportionately drive tissue‐level inflammatory signals with age. Overall, these findings position inflammaging as a cell‐type‐specific and cell state‐dependent process that is tightly linked to broader aging‐associated changes such as cellular senescence.

## 3 Cell–Cell Communication During Arterial Aging

Arterial function relies on cell–cell signaling across the vessel wall, where ECs, SMCs, and adventitial fibroblasts exchange signals to control vascular tone and coordinate remodeling after stress (Méndez‐Barbero et al. 2021). Immune cells, such as macrophages, are recruited by chemokine signals released from SMCs and contribute to vascular remodeling by amplifying local inflammation, supporting a key role for cell–cell communication in the arterial wall (Qi et al. 2019). With aging, intercellular signaling in the arterial wall shifts toward endothelial dysfunction, oxidative stress, and chronic low‐grade inflammation (Méndez‐Barbero et al. 2021). The arterial wall also remodels at the structural level, with changes of collagen and elastin proportions and properties that raise stiffness and alter how vascular cells sense force (Qi et al. 2019). SMCs respond by changing phenotype, which supports maladaptive remodeling and can enable osteogenic‐like programs linked to calcification (Lanzer et al. 2021, 2025). Adventitial fibroblasts can contribute to “outside‐in” inflammatory signaling, where fibroblast‐derived cytokines such as IL‐6 promote SMC proliferation and migration after injury (Dutzmann et al. 2025). In parallel, fibroblast‐specific Nox2–dependent signaling has been shown to promote paracrine signaling to SMCs via factors such as GDF6, thereby driving SMC growth and contributing to vascular remodeling (Harrison et al. 2021). Interlayer signaling can also be mediated by extracellular vesicles (EVs): EC–derived EVs can induce inflammatory and senescent features in SMCs, whereas EV biogenesis within SMCs contributes to calcification processes that can be attenuated by epidermal growth factor receptor (EGFR) inhibition in experimental models (Bakhshian Nik et al. 2023; Boyer et al. 2020; Lanzer et al. 2025).

An important recent single‐cell transcriptomic study by Xie et al. provides additional support for the role of altered intercellular communication in vascular aging. In the mouse aorta, communication among vascular cells declines while immune‐related interactions increase, with reduced EC–SMC signaling and enhanced EC–monocyte interactions suggesting a shift toward inflammatory activation (Xie et al. 2022). ECs also exhibit dysregulated signaling programs during aging, characterized by increased communication among angiogenic and proliferative EC subsets but reduced immune‐related pathways such as tumor necrosis factor (TNF) and CC chemokine ligand (CCL) signaling (Liu et al. 2023). However, in primate aorta datasets, EC–SMC signaling is enhanced, particularly in pathways linked to vascular remodeling and calcification, including Notch, PDGF, BMP, and EGF signaling, with Betacellulin–EGFR interactions highlighted as potential drivers of vascular aging (Yin et al. 2022).

Vascular aging is increasingly understood as a coordinated reprogramming of intercellular communication networks across the arterial wall, involving endothelial dysfunction, smooth muscle cell plasticity, fibroblast activation, and immune cell recruitment. Rather than reflecting a uniform change, these interactions are dynamically reshaped toward inflammatory and remodeling‐associated signaling. Future studies that spatially resolve the effects of functional perturbations of ligand–receptor interactions will be essential for causal resolution of the cellular context of immune‐vascular interactions and to identify key signaling pathways that govern vascular aging.

## 4 Cellular Contributors to Arterial Aging

The following section synthesizes how major vascular cell types contribute to arterial aging with a focus on the phenotypic aging hallmarks introduced above. To improve cross‐study comparison and reduce inconsistencies in nomenclature, we organize vascular cell states across major vascular cell types based on shared functional programs, rather than relying solely on study‐specific cluster labels. Across ECs, SMCs, and fibroblasts, aging‐associated phenotypes frequently converge on recurring functional features, including senescence‐associated, inflammatory, and extracellular matrix remodeling‐related states. Although the exact transcriptional signatures and relative abundances vary across studies and species, this functional perspective provides a useful framework to interpret cellular heterogeneity during arterial aging.

### 4.1 Endothelial Cells

ECs lining the intima layer act as a critical barrier in the arterial wall, regulating vascular tone, preventing thrombosis, and maintaining vascular homeostasis by releasing vasodilators like nitric oxide (NO) and prostacyclin, while also producing anti‐inflammatory and antithrombotic factors. Thus, ECs prevent CVDs such as atherosclerosis by maintaining blood fluidity and inhibiting platelet aggregation (Boulanger 2018; Hirase and Node 2012; Indranil and Gausal 2019). During arterial aging, ECs shift from a vasoprotective to a dysfunctional state. This includes lower NO signaling, higher oxidative stress, and increased adhesion molecules that promote leukocyte recruitment (Akhiyat et al. 2025; Herzog et al. 2025; Ungvari et al. 2018). Endothelial dysfunction also supports ECM remodeling that contributes to arterial aging (Herzog et al. 2025).

**FIGURE 1:**
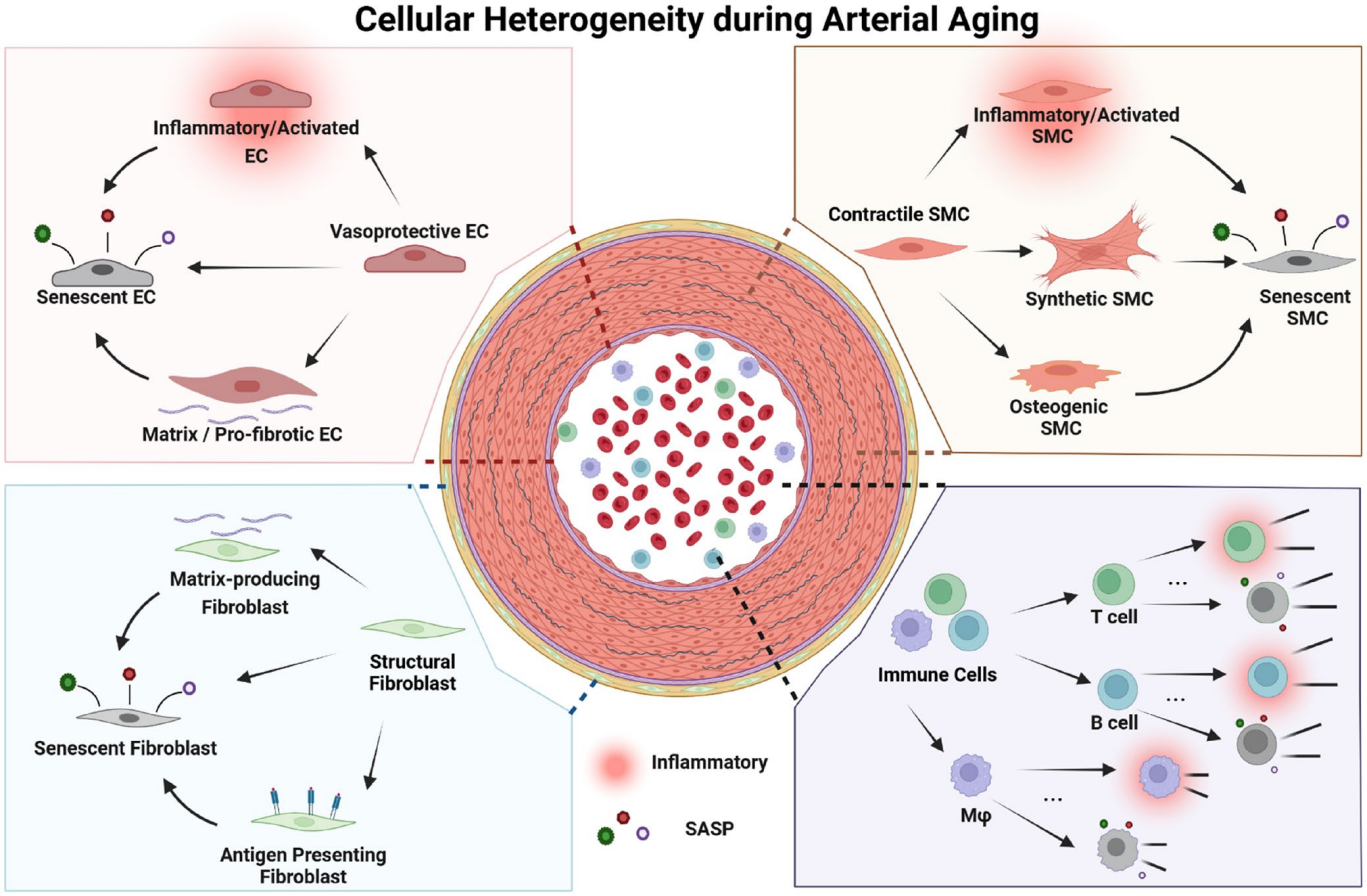
Graphical abstract showing arterial cell subpopulations and their functional changes during aging. Transcriptomic profiling studies across species identify age‐associated endothelial, smooth muscle, fibroblast, and immune cell states characterized by inflammation, extracellular matrix remodeling, phenotypic switching, and cellular senescence. Homeostatic endothelial cells of the intima acquire pro‐inflammatory and pro‐fibrotic phenotypes, while contractile smooth muscle cells in the media transition toward activated, synthetic, and osteogenic states. Adventitial fibroblast increase matrix production, and can acquire antigen presenting properties through major histocompatibility complex class II (MHC II) expression. Immune cells become highly heterogenous in response to inflammatory stimuli and antigen exposure. Persistent cellular stress, including reactive oxygen species (ROS) mediated damage or increased cell turnover, forces arterial cells into premature or replicative senescence. Detailed expression profiles contributing to arterial cell heterogeneity during natural aging and in the presence of associated risk factors are listed in Tables 1 and 2.

Single cell transcriptomic studies have identified multiple EC clusters in vascular aging. To facilitate clarity and cross‐study comparison, we propose a unified nomenclature for aging‐associated EC subtypes, categorizing them into senescent ECs, matrix‐remodeling ECs, inflammatory ECs, and vascular homeostatic ECs (Figure 1). These subtypes have been repeatedly identified across multiple aging datasets in mice and nonhuman primates, underscoring a degree of evolutionary conservation. Despite some interspecies differences in cluster naming and granularity, the recurrence of these EC phenotypes in both mouse and monkey datasets suggests that key features of endothelial aging are conserved across mammals.

These functionally defined EC states are supported by multiple single‐cell studies across species. Under physiological conditions, homeostatic ECs (vascular tone–regulating ECs) maintain vascular function through regulation of nitric oxide production, vasodilation, and endothelial barrier integrity. However, with aging, there is a shift in EC state composition toward senescent and inflammatory phenotypes. For example, in a study of young and aged female mouse hearts, Liu et al. identified ten EC subclusters, including capillary, capillary arterial, capillary venous, arterial, venous, and lymphatic endothelial populations, as well as angiogenic, proliferating, and immunology‐associated EC states. The authors showed that ECs from aged mice exhibit significantly higher senescence‐associated secretory phenotype (SASP) and inflammation gene‐set scores. (Liu et al. 2023). This is in line with findings that suggest endothelial dysfunction in aging and thus susceptibility to age‐related CVDs, is largely attributed to EC senescence (Han and Kim 2023).

Consistently, Xie et al. used scRNA‐seq and scATAC‐seq in male mice aged 4, 26, and 86 weeks to identify two endothelial subpopulations, termed EC1 and EC2, that were present across all ages. Rather than representing age‐specific clusters, EC1 exhibited progressively increased gene set activity associated with cellular senescence, SASP, and inflammation with advancing age. This was supported by experimental validation showing higher SA‐β‐gal staining, increased P53 and P21 protein levels, and reduced Ki67 expression in EC1 compared with EC2. EC1 was further characterized by high expression of genes such as *Vcam1*, *Cd36*, *Rgcc*, and *Gpihbp1*. In contrast, EC2, marked by *Plvap* and *Vwf* expression, was associated with vascular tone regulation through Nos3, Edn1, and Ace expression and increased NO production (Xie et al. 2022). This was further substantiated by in vivo findings indicating higher vasodilatory response in certain aortic segments enriched with Plvap^+^ ECs. Importantly, by separating the senescent EC cluster along a pseudotime trajectory that distinguished young, 4 and 26 weeks, from old, 86 weeks, mice, the authors further assessed motif activity and transcription factor expression using the accompanying scATAC‐seq data and identified TEA domain family member 1 (Tead1) as one candidate regulator (Xie et al. 2022).

Importantly, these endothelial states are not unique to aging but appear to reflect pre‐existing endothelial heterogeneity. Consistent with this, single‐cell analysis of the normal mouse aorta identified three endothelial subpopulations already present under physiological conditions, including populations specialized in extracellular matrix production, lipoprotein handling, and angiogenesis. (Kalluri et al. 2019). Additionally, re‐analysis of published scRNA‐seq from cynomolgus monkey aortae revealed an EC subtype that exhibited high expression of genes involved in ECM remodeling, such as *FN1, COL3A1, BMP4*, *SPARC*, which are associated with vascular calcification and arterial stiffening during aging (Yin et al. 2022; Zhang et al. 2020). BMP4 has been involved in SMC calcification and is upregulated in atherosclerotic lesions (Dhore et al. 2001; Hayashi et al. 2006; Mikhaylova et al. 2007), while *SPARC* (encoding for osteonectin) and *FN1* (encoding for fibronectin) are ECM molecules associated with calcification or fibrosis, potentially exacerbating arterial stiffening (Ciceri et al. 2016; Hoeft et al. 2023).

Beyond the identification of EC states, several studies have begun to investigate regulatory mechanisms associated with endothelial aging. Across the studies discussed, EC clusters were often annotated using gene set activity or gene ontology enrichment as proxies for aging, rather than being directly defined based on chronological age groups. This approach constrains biological interpretation, as it does not directly capture age‐dependent shifts in EC populations based on real biological age. TEAD transcription factors are the main co‐factors of YAP/TAZ transcription factors, which are part of the highly mechano‐sensitive Hippo pathway (Piccolo et al. 2014). As the mechanical properties of the ECM drastically change during aging and YAP/TAZ signaling has been shown to guard SMCs from acquiring a senescent phenotype (Sladitschek‐Martens et al. 2022), investigation of YAP/TAZ and Tead TFs in age‐induced senescence of other vascular cells like ECs and fibroblasts represents an intriguing avenue for future research. Consistent with this hypothesis, Liu et al. showed that YAP is elevated in senescent endothelial cells and that inhibition of YAP attenuates EC senescence in vitro (Liu et al. 2025). Furthermore, this highlights that not only the age‐dependent transcriptional adaptations themselves, but also their upstream regulators are important to investigate. In this regard, the transcription factor BTB and CNC Homology 1, Basic Leucine Zipper Transcription Factor 1 (*BACH1*) has been identified as a key regulator of aging‐associated gene expression in ECs of coronary arteries and aortic arches of cynomolgus monkeys as well as the murine cardiac vasculature (Ge et al. 2021). BACH1 expression was observed to be upregulated in vascular ECs of aged mice in vivo, as evidenced by immunohistochemistry (IHC) analysis of mouse aortae. Interestingly, knockdown of *BACH1* significantly inhibited H_2_O_2_ induced senescence in human umbilical vein endothelial cells (HUVECs), while overexpression promoted senescence. Mechanistically, BACH1 promoted EC senescence by binding to enhancer regions of the *CDKN1A* gene, thereby driving the transcription of P21, a key effector in cell cycle arrest and senescence (Ge et al. 2021). A separate study on cynomolgus monkeys underscored the pivotal role of the transcription factor *FOXO3A* in EC aging by applying gene regulatory network analysis on EC clusters of young and aged monkeys identified by scRNA‐seq (Zhang et al. 2020). Functionally, they could show that FOXO3A downregulation in vitro in human arterial and venous cells recapitulated age‐associated phenotypes, that is, impaired proliferation and migration of ECs. FOXO3A loss with aging in vivo might thus disrupt vascular homeostasis and heightens the risk of cardiovascular diseases, such as atherosclerosis (Zhang et al. 2020).

Endothelial‐to‐mesenchymal transition (EndoMT) adds another layer of EC plasticity and contributes to atherosclerosis, including in premature‐aging models (Hamczyk et al. 2024; Souilhol et al. 2018). Single‐cell and lineage‐informed analyses indicate that EndoMT does not form a discrete mesenchymal cluster but instead represents a continuum of mesenchymal activation across EC subtypes, often appearing as partial or intermediate states (Lebas et al. 2024; Slenders et al. 2025). Trajectory analyses in human atherosclerotic tissue further show that EndoMT‐associated programs emerge progressively along pseudotime, with mid‐stage signatures reflecting active transition rather than terminal differentiation (Slenders et al. 2025). This overlapping transcriptional state complicates cell type annotation in scRNA‐seq datasets, as transitioning cells retain endothelial features while acquiring mesenchymal traits. Mechanistically, mitochondrial Ca^2+^ uptake is required for EndoMT, and its inhibition prevents mesenchymal activation in vitro and in vivo (Lebas et al. 2024).

Together, these findings provide the cellular basis for future studies aimed at age‐dependent vascular heterogeneity in humans and highlight conserved endothelial states, including senescent, inflammatory, matrix‐remodeling, and homeostatic ECs, that may serve as therapeutic targets across species.

### 4.2 Smooth Muscle Cells (SMCs)

SMCs, located in the media layer, provide structural support, regulate blood vessel contraction, and are responsible for maintaining vascular tone through intercellular communication with ECs (Hu et al. 2021). They synthesize key ECM elements like elastin and collagen that preserve arterial elasticity and structural integrity (Kielty et al. 2007; Krajnik et al. 2023; Reed et al. 2022). With aging, SMCs shift from a contractile to a synthetic phenotype, driven by oxidative stress, inflammation, and an imbalance between anti‐ and proaging factors (Chan and Fiscus 2004; Hoffmann et al. 1998; Lacolley et al. 2017). This leads to excessive ECM deposition, particularly collagen, contributing to arterial stiffness. While these phenotypic changes have been well‐documented at the cell type level, recent scRNA‐seq studies have revealed greater heterogeneity within the SMC population, providing new insights into the molecular mechanisms driving vascular aging. Across species, single‐cell analyses have identified conserved SMC subpopulations associated with vascular aging, including synthetic, proliferative, apoptotic, and stressed/inflammatory phenotypes (Figure 1). Synthetic SMCs, representing a homeostatic SMC state, are marked by *Spp1* and *Fn1*. These have been consistently observed in aged vessels of rats, mice, and monkeys, and appear to persist or even expand with age. In contrast, proliferative SMCs, identified by markers such as *Ccl2*, *Ccl7*, and *Procr*, show a notable decline during aging in both rats and mice. Evidence from monkey and human datasets further supports shared transcriptional programs involving inflammatory and ECM‐remodeling pathways. These conserved SMC states suggest common mechanisms of phenotypic modulation in vascular aging.

A recent study, using male Wistar Kyoto rats (WKY) as normotensive controls for a hypertension model, has provided detailed insights into the phenotypic shift of aging SMCs using scRNA‐seq. In the aortae of both young and aged rats they identified synthetic (*Spp1* ^+^, *Pam* ^+^, *Dcxr* ^+^, *Fbln5* ^+^), stressed (*Egr1*/*2* ^+^, *Fos* ^+^, *Jun* ^+^), apoptotic (*Xirp1* ^+^, *Mt1* ^+^, *Sphk1* ^+^, *Sdc4* ^+^), and proliferative SMCs (*Ccl7* ^+^, *Gsn* ^+^, *Ccl2* ^+^. *Procr* ^+^) (Cheng et al. 2024). Based on pathway enrichment analysis and gene expression the authors suggest that aged SMCs show reduced contractility but increased inflammation and senescence—effects that were intensified when aging was superimposed with hypertension (Cheng et al. 2024). Importantly, the authors identified *Spp1* (osteopontin) as a marker for synthetic SMCs and could show that *Spp1* is markedly increased in α‐SMA positive SMCs in hypertensive aging (Cheng et al. 2024). This is in line with previous results highlighting that arteries of hypertensive rats are stiffer than those of WKY rats which has been attributed to extracellular dysfunction of SMCs (increased collagen secretion and collagen fibril disorganization) and was accompanied by a diminished adaptability to stretch of SMCs (Hays et al. 2018). Similarly, SMCs of aged mice show defects in mechano‐sensation compared to those of younger mice (Luu et al. 2024). Interestingly, sex‐stratified bulk RNA sequencing data from human CVD studies combined with lineage tracing scRNA‐seq data revealed key differences in SMC phenotype switching, with female‐specific drivers of gene regulatory networks like *GAS6* and *FN1* being expressed primarily in SMCs that lost their traditional markers (Hartman et al. 2021), suggesting that sex‐specific factors influence SMC plasticity in atherosclerosis and highlighting the need to investigate sex‐specific differences for other age‐related CVDs. In cynomolgus monkeys, pathway enrichment analysis of differentially expressed genes between young and ages vessels indicated that aging‐associated alterations in SMCs include changes in inflammatory response and ECM organization (Zhang et al. 2020). A study investigating age‐related changes in the murine ascending thoracic aorta found that SMCs display enhanced β‐galactosidase hydrolysis, indicating increased senescence‐associated features during aging (Rivera et al. 2024). However, rather than defining a discrete senescent SMC state, the authors identified two VSMC subsets, one that declined markedly with aging and was associated with proliferative and regenerative programs, and another that was relatively preserved and displayed a more secretory phenotype. They identified two distinct SMC subsets, one of which drastically declined during aging (Rivera et al. 2024). Based on pathway enrichment analysis, they characterized the declining subset as proliferating SMCs, while the subset that remained stable in aging was characterized as synthetic (Rivera et al. 2024).

Together, these studies consistently identify a set of conserved SMC states across species and experimental conditions, including synthetic, proliferative, apoptotic, and stressed/inflammatory phenotypes. These recurrent states suggest that SMC phenotypic modulation during aging follows shared transcriptional programs rather than representing entirely distinct, study‐specific populations. However, the mechanisms underlying SMC‐specific responses to aging, particularly in different vascular beds, and the interplay of these responses with comorbidities such as hypertension or hypercholesterolemia are not fully understood.

### 4.3 Fibroblasts

Fibroblasts, residing primarily in the adventitial layer, contribute to ECM production and structural stability (Michel et al. 2022). Fibroblasts are primarily responsible for synthesizing collagen (particularly types I and III), critical components of the ECM in the adventitia (Stenmark et al. 2013). Fibroblasts continuously remodel the ECM in response to mechanical stress and biochemical signals, increasing the production of collagen and proteoglycans, which are key to sustaining vascular integrity (Osherov et al. 2011; Stenmark et al. 2013). It has been shown that when collagenase is used, the stiffness of the adventitia is reduced 45‐fold, emphasizing the key role of collagen produced by fibroblasts (Beenakker et al. 2012). With aging, increased remodeling of the ECM and inflammation accelerate arterial stiffness and functional decline. In particular, adventitial collagen accumulation precedes the onset of medial fibrosis suggesting the involvement of adventitial fibroblasts (Longtine et al. 2024; van Kuijk et al. 2023). In addition, fibroblasts are essential in vascular injury repair. In response to damage, they can transition into myofibroblasts, which secrete additional ECM proteins to facilitate tissue repair (Zalewski and Shi 1997) and are marked by *ACTA2* expression (Fleenor et al. 2010).

In the aged murine ascending thoracic aorta, fibroblasts upregulated collagen‐related genes, including *Col1a1*, *Col1a2*, *Col3a1*, and *Col6a2*, which was accompanied by increased collagen deposition in the media and aortic stiffening (Rivera et al. 2024). Similarly, adventitial collagen content significantly increased in carotid arteries of old (29–32 month) compared to young (4–7 months) mice, accompanied by an increase in smooth muscle actin (*ACTA2*) positive cells in the adventitia, indicating enrichment of myofibroblasts (Fleenor et al. 2010). While there is evidence for the importance of collagen deposition in vascular aging and adventitial fibroblasts have been identified as major collagen‐producing cells, no experimental studies have directly demonstrated the causal role of fibroblasts or their collagen production in vivo using fibroblast depletion or gene silencing approaches.

In single‐cell RNAseq studies of the mammalian vasculature, we and others identified distinct fibroblast subsets which can be synthesized as follows: ECM remodeling fibroblasts, progenitor fibroblasts, and inflammatory fibroblasts (Figure 1).

At the cross‐tissue level, broader fibroblast heterogeneity has been described. A cross‐tissue fibroblast scRNA‐seq‐based atlas identified ten distinct fibroblast subsets in mice (Buechler et al. 2021). In particular, *Pi16* ^+^ and *Col15a1* ^+^ fibroblast subtypes were present surrounding vasculature in all organs and tissues and broadly distributed across both homeostatic and pathological states. Their gene expression pattern resembled fibroblast subsets found in humans (Buechler et al. 2021). Based on lineage inference analysis, the authors suggested that *Pi16* ^+^ fibroblasts might serve as a progenitor cell capable of differentiating into specialized, organ‐specific fibroblasts, while they associated the *Col15a1* ^+^ subtype with the ability to secrete basement membrane proteins based on expressed genes. However, the authors did not specifically address the functional changes in arterial fibroblasts during the aging process. We have previously performed scRNA‐seq analysis in healthy and diseased murine and human arteries and identified three subpopulations of fibroblasts marked by *CD55*, *CXCL14*, and *LOX* that displayed functional differences in different trajectories (van Kuijk et al. 2023). In detail, *CD55* ^+^ and *CXCL14* ^+^ fibroblasts were particularly associated with collagen accumulation and adventitial thickening, while LOX+ fibroblasts were associated with collagen crosslinking‐related gene expression and coincided with increased collagen deposition and arterial stiffening (van Kuijk et al. 2023). The plasticity of fibroblasts, including their ability to express stem cell markers like *Sca‐1/Ly6a*, allowed them to switch between functional states, contributing to both tissue repair and disease progression in aging arteries (van Kuijk et al. 2023). Xie and colleagues subsetted their scRNA‐seq data from the aortae of young and aged mice for fibroblasts and used a pseudotime‐based approach to distinguish two cell fates that accumulated in aged mice and a pre‐state primarily present in young mice (Xie et al. 2022). Based on gene ontology enrichment analysis, the authors identified two fibroblast populations: one enriched for pathways related to connective tissue development, muscle cell proliferation, and muscle adaptation, and another characterized by high expression of genes associated with collagen metabolism and immunoinflammatory processes (Xie et al. 2022). Using matching scATAC‐seq data, the authors further investigated transcription factor activity specific for each cell fate. For the proliferative subset, they associated expression and enhanced promoter motif activity of *Stat3*, *Nfil3*, and *Hoxa5* with senescence (Xie et al. 2022). For the immunoinflammatory subset, they showed high expression and motif activity of *Creb5* (Xie et al. 2022), which has been associated with inflammaging in humans (Nevalainen et al. 2015).

Using cynomolgus monkey data, Zhang et al. observed that adventitial fibroblasts exhibited a significant increase in transcriptional noise during aging, indicating greater instability in gene expression as the cells aged (Zhang et al. 2020). Aging fibroblasts showed changes in gene expression associated with inflammatory responses, lipid metabolism, and calcium signaling pathways. Additionally, the authors identified a specific mRNA marker phosphatidylethanolamine‐binding protein 4 (*PEBP4*) for adventitial fibroblasts. However, based on the provided cell classification and the expression of *PEBP4* among cell clusters, it seems that *PEBP4* is also expressed in SMCs, although to a lesser extent (Zhang et al. 2020). Moreover, its expression in intact tissue on protein level was not studied (Zhang et al. 2020). These studies reveal consistent findings on the role of adventitial fibroblasts in arterial stiffness and ECM remodeling; however, differences in aging patterns and inflammatory responses exist. For instance, the priorly referenced study of Xie et al. highlighted increased transcriptional noise and upregulation of proinflammatory pathways (e.g., TNF‐α, NF‐κB) with age (Xie et al. 2022). Although primate models also show increased transcriptional noise as fibroblasts age, they also reflect additional changes in lipid metabolism and calcium signaling pathways, reflecting a potentially more complex age‐related phenotype (Zhang et al. 2020). These interspecies variations indicate that while the fundamental role of fibroblasts in arterial aging is shared, certain molecular pathways and susceptibility to stimuli may be species‐specific.

Importantly, sex differences in aging‐associated fibroblast function and subset specification remain unresolved. Zhang et al. included equal numbers of male and female mice (*n* = 4 per group) for young and aged groups, but the analysis did not explicitly stratify results by sex. Moreover, the study by Xie et al. focused exclusively on male mice to avoid transcriptomic alterations due to differences in sex hormone expression upon menopause entry. This highlights the need for future research to incorporate sex‐stratified analyses to deepen our understanding of fibroblast behavior across sexes and its implications in cardiovascular disease. For this, we will need larger cohorts including equal numbers of female and male animals (or humans) and need to find ways to account for age‐related changes in sex hormone secretion.

Despite the findings described in this review, the research on fibroblast alterations during arterial aging remains relatively sparse compared to other vascular cell types. While fibroblasts play a crucial role in arterial stiffening and fibrosis in hypertension, their contributions in vascular aging are not as thoroughly investigated as ECs or SMCs (Guzik and Mikolajczyk 2014). The potential of fibroblasts to transition into myofibroblasts, likely driving ECM remodeling and inflammation, underscores their potential importance in vascular aging. Also, many aspects of their involvement, particularly the molecular mechanisms governing their plasticity and response to aging stimuli, are not yet fully understood.

### 4.4 Immune Cells

In arteries, immune cells play a pivotal role in maintaining vascular function and regulating inflammation. As aging progresses, immune cell function gradually declines, leading to inflammaging (Ajoolabady et al. 2023). Rather than being solely defined by changes in cell proportions, immune aging is increasingly recognized as a shift toward distinct functional and transcriptional states across immune cell populations. Importantly, there are extensive datasets investigating immune aging in humans by profiling peripheral blood mononuclear cells (Filippov et al. 2024; Gong et al. 2025), but data on tissue resident and infiltrating immune cells remain rather sparse. Here, we aim to group recent findings on immune cells from single cell studies of arterial aging along the key phenotypic hallmarks of vascular aging (inflammaging, immuno‐senescence).

During aging, both human and mouse macrophages have been reported to adopt a more proinflammatory phenotype, often reflected by enhanced TNF and IL‐6 production, particularly after ex vivo stimulation, although these responses vary across tissues and macrophage populations. (van Beek et al. 2019). These proinflammatory macrophages contribute to persistent inflammatory responses, particularly in CVDs (Barcena et al. 2022). In this process, reparative macrophage functions decline, as indicated by reduced expression of *Arg1* and *Sod3* (Haschak et al. 2021), suggesting an imbalance between inflammatory and tissue‐resolving programs. In arterial aging contexts, single‐cell analyses further identified transcriptionally distinct macrophage states, such as *Spp1* ^high^ scar‐associated macrophages, which are expanded in aged vessels and enriched for genes related to inflammation, extracellular matrix remodeling, and oxidative stress (Cheng et al. 2024). These findings suggest that macrophage‐driven inflammaging may be mediated by specific molecular programs rather than uniform activation.

A similar shift toward functionally altered states is observed in T cells. As arteries age, CD4^+^ and CD8^+^ T cells exhibit features of senescence, including reduced proliferative capacity and altered cytokine secretion (Pan et al. 2020). At the single‐cell level, distinct T cell states emerge during aging. For example, GzmK^+^ CD8^+^ T cells display combined cytotoxic and exhaustion‐like features, expressing effector molecules such as Gzmk and Prf1 alongside inhibitory markers including Pdcd1 and Lag3 (Smit et al. 2023). In humans, aging is associated with accumulation of proinflammatory memory T cell populations, including GZMK‐expressing CD8^+^ effector memory T cells and HLA‐DR^+^ CD4^+^ memory T cells, as well as type‐2–skewed CD4^+^ T cells producing IL‐4 (Terekhova et al. 2023). Together, these findings suggest that T cell aging is characterized not only by senescence but also by functional reprogramming toward proinflammatory and dysregulated immune states.

B cells also undergo age‐associated molecular changes that contribute to vascular inflammation. Age‐associated B cells (ABCs), characterized by CD11b^+^CD11c^+^T‐bet^+^ phenotypes, are expanded in aged arteries and exhibit enhanced antigen‐presenting and proinflammatory capacities (Smit et al. 2023) and are dependent on Zeb2 transcriptional programs (Dai et al. 2024). These cells can activate T cells through costimulatory signaling (e.g., Cd40, Cd80, Cd86), thereby amplifying local immune responses and potentially contributing to plaque progression. Interestingly, aged B cell‐depleted mice display reduced CD4 T cell immune‐senescence compared to control mice, identifying B cells as critical mediators that drive age‐associated adaptive immune dysfunction (Khan et al. 2026).

In addition to these molecularly defined immune states, changes in immune cell composition are also observed during arterial aging. Xie et al. reported that increased proportions of neutrophils, mast cells, and plasmocytes, together with reduced dendritic cells, have been reported (Xie et al. 2022). However, these compositional changes are accompanied by substantial transcriptional heterogeneity and altered cytokine signaling, including increased IL‐1β, IL‐6, and IL‐10 expression (Xie et al. 2022), suggesting that functional reprogramming occurs alongside shifts in cell abundance.

Together, these findings support a model in which immune aging in arteries is driven by the emergence of functionally distinct, proinflammatory and dysregulated immune cell states, rather than solely by changes in cell proportions. Across species, these include proinflammatory macrophage subsets, cytotoxic and exhaustion‐associated T cell populations, and antigen‐presenting B cell states, which collectively contribute to chronic inflammation, impaired immune regulation, and vascular remodeling. Understanding these molecular programs will be essential for developing targeted interventions against age‐related vascular diseases. Moreover, the influence of sex on these immune‐mediated mechanisms remains poorly understood, as most studies focus predominantly on male physiology.

## 5 Cross Species Insights From Single Cell Studies

Cross‐species comparisons provide an important framework for interpreting single‐cell studies of vascular aging. While many aging studies focus on changes within a single species, natural lifespan varies dramatically across mammals, suggesting that mechanisms associated with longevity cannot be fully understood from one model alone (Ma and Gladyshev 2017; Tyshkovskiy et al. 2023). Comparative studies further indicate that longevity is shaped by both conserved and lineage‐specific programs, and that mechanisms associated with naturally long‐lived species are only partly overlapping with those identified from lifespan‐extending interventions within species (Ma and Gladyshev 2017; Tyshkovskiy et al. 2023). Single‐cell studies indicate that arterial aging is driven by partially conserved cellular programs across species. In mice, rats, nonhuman primates, and humans, aging is repeatedly associated with endothelial dysfunction, SMC phenotypic switching, fibroblast‐mediated matrix remodeling, and immune activation. The main cross‐species commonality lies in the recurrence of inflammatory, senescent, and ECM‐remodeling states, whereas the major differences concern subtype resolution, relative cell abundance, and the specific regulatory pathways highlighted in each model. Thus, current evidence supports conservation of core aging‐associated vascular cell states but also suggests that their molecular execution is shaped by species, vascular bed, sex, and disease context. These observations highlight the need for systematic cross‐species integration to distinguish conserved programs from context‐dependent adaptations. Cross‐species integration in vascular biology is no longer just a theoretical idea, but current studies are still fragmented. Transcriptome‐level comparison across rats, monkeys, and humans has shown that conserved vascular aging signals do exist, particularly in matrix‐ and adhesion‐related pathways, yet many canonical inflammatory and senescence‐associated programs are not uniformly regulated across species, limiting straightforward extrapolation from animal models to humans (Sun et al. 2024). Complementing this, integrated single‐cell analysis of mouse and human atherosclerosis has shown that several mononuclear phagocyte states are transcriptionally conserved across species, providing evidence that at least part of lesion‐associated immune heterogeneity is evolutionarily preserved (Zernecke et al. 2023).

Thus, while cross‐species vascular studies have begun to identify either conserved pathways or conserved immune‐cell states, a comprehensive framework linking conserved and species‐specific aging programs across EC, VSMCs, fibroblast, and immune compartments is still missing.

## 6 Future Perspectives

Despite recent advances, several unresolved questions remain. A key challenge is the functional validation and causal roles of subpopulations identified through scRNA‐seq. While the studies summarized in this review have provided valuable insights into cellular heterogeneity, the precise roles of the identified subpopulations were not fully elucidated. One point to address is that in several studies authors did not show changes in cell type composition depending on age or only show composition changes in supplementary figures without any comments. It would be of major interest if subtype proportions change with age and if not, if there are differences in genes expressed in young and aged groups. Furthermore, future research should focus on in vivo models and functional assays, including gene‐editing techniques like CRISPR/Cas9, to confirm the causal roles of these cell subsets in (arterial) aging. Meta‐analyses of scRNA‐seq datasets across species, including humans, mice, and nonhuman primates, could further clarify conserved pathways in arterial aging and improve the translational relevance of animal studies to human aging.

A crucial area for future research is the exploration of sex‐specific differences in arterial aging at the single‐cell level. While recent advances in scRNA‐seq have deepened our understanding of cellular heterogeneity, relatively little is known about how these cell populations and their roles may differ between males and females. Future studies should aim to incorporate both male and female models to identify sex‐specific transcriptomic and epigenetic profiles. This approach could provide valuable insights into distinct aging mechanisms and help develop more personalized strategies for preventing or treating vascular dysfunction in aging populations. In addition, the limited availability of human aging‐specific/nondiseased arterial data largely reflects technical challenges associated with tissue dissociation, particularly in fibrotic or calcified vascular samples. Single‐nucleus RNA sequencing (snRNA‐seq) provides an important complementary approach by enabling transcriptomic profiling from frozen or hard‐to‐dissociate tissues. This approach may therefore facilitate transcriptomic profiling of human vascular tissues that are otherwise difficult to analyze, enabling more comprehensive characterization of arterial aging and improving cross‐species comparability in future studies. Along the same way, new technical developments now allow the use of archived tissue samples for transcriptional profiling (e.g., 10× FLEX), facilitating complex sample processing routes for human vascular samples.

Moreover, transcriptomics analysis does not capture epigenetic modifications, such as DNA methylation and chromatin accessibility, which are critical regulators of gene expression during aging. Integrating scRNA‐seq with epigenetic techniques, such as scATAC‐seq or DNA methylation profiling, could provide a more comprehensive understanding of the molecular underpinnings of vascular aging. A key limitation of most arterial aging scRNA‐seq datasets is that dissociation removes spatial context, so we cannot reliably place aging‐associated subpopulations within the layered vessel wall or within specific microenvironments (e.g., fibrous, inflamed, or calcifying regions). Spatial transcriptomics can address this by mapping cell states and their signaling programs back to histologically defined niches. Recent vascular studies highlight the value of spatial approaches: spatially resolved analyses of human atherosclerotic plaques have revealed region‐specific cell–cell communication and identified a fibroblast‐like vascular smooth muscle cell state that functions as a signaling hub associated with plaque stability (Goncalves et al. 2026). Spatial approaches also localize senescence‐associated vascular programs and remodeling niches in mouse atherosclerosis and aneurysm models (Mazan‐Mamczarz et al. 2025). Integration of single‐cell and spatial data using deep learning models such as cell2location and Tangram can infer fine‐grained cell‐type maps in situ and link cell states to local microenvironments (Kleshchevnikov et al. 2022). Looking forward, development in artificial intelligence like single‐cell foundation models (e.g., scGPT) may help automate and harmonize cross‐study vascular datasets and prioritize conserved aging programs for targeted spatial and functional validation (Cui et al. 2024).

With the advent of single cell sequencing, it has become increasingly apparent that chronological age and biological age are sometimes disconnected and that particular cell types have a higher biological age than others. To evaluate this, researchers have introduced “aging clocks” that might help to evaluate the effect of targeted interventions on cellular age. However, the results of different clocks show rather low degrees of correlation (Spray et al. 2025). Thus, improving and harmonizing these aging clocks could help resolve cellular contributions to vascular aging.

Furthermore, exploration of cell–cell communication is crucial, being a hallmark of aging. Aging impacts not only individual cells, but also the signaling networks between them. Investigating how intercellular signaling evolves with age could provide new insights into the coordination of vascular responses to aging stimuli. Additionally, examining the effects of lifestyle factors—such as diet, exercise, and stress—on arterial aging at the single‐cell level could provide valuable insights into how external factors modulate cellular and molecular pathways associated with aging. Based on these insights, future research should focus on integrating multidisciplinary approaches to further clarify the cellular and molecular mechanisms of arterial aging, ultimately guiding the development of targeted therapeutic strategies to mitigate vascular dysfunction and promote healthy aging.

## 7 Conclusion

The emergence of scRNA‐seq has enabled high‐resolution characterization of cellular heterogeneity during vascular aging, revealing a broad spectrum of EC, SMC, and fibroblast subpopulations. However, as in other single‐cell–driven fields, cell states are often defined by study‐specific gene expression signatures rather than shared functional or phenotypic patterns, which limits cross‐study comparability and interpretability. To address this, we proposed a harmonized nomenclature for major vascular cell types that emphasizes conserved functional states along key aging‐related phenotypic programs (Figure 1). For ECs, we distinguish senescent, matrix‐remodeling, inflammatory, and vascular tone‐egulating phenotypes; for fibroblasts, ECM‐remodeling, progenitor‐like, and inflammatory states, with senescence‐associated features reported but not clearly resolved as a discrete fibroblast subset; for SMCs, synthetic, proliferative, apoptotic, and stressed/inflammatory phenotypes, with senescence likewise observed mainly as a functional overlay rather than a distinct transcriptional state; and for immune cells, aging is characterized by functionally distinct but lineage‐spanning states, including proinflammatory macrophage states, cytotoxic and exhaustion‐associated T‐cell states, and antigen‐presenting B‐cell populations, whereas senescent immune subsets have not been consistently or explicitly defined. To provide a structured overview of the current landscape and to contextualize this proposed framework, we systematically summarized representative single‐cell studies of vascular aging in Table 1 (natural aging) and Table 2 (aging with additional risk factors). We anticipate that this framework will improve the integration and reproducibility of both existing and future datasets and provide a coherent reference that facilitates communication and discovery within the vascular aging research community.

**TABLE 1:** Summary of single‐cell transcriptomic studies on cellular heterogeneity in natural arterial aging.

Study	Species	Ages	Gender	Artery/Microvascular site	Subsets	Markers
Yin et al. (2022), Zhang et al. (2020)	*Cynomolgus* monkey^a^	Young (4–6 years), Old (18–20 years)	Male and Female	Aorta and Coronary Artery	EC subpopulations related to ECM remodeling and calcification	*FN1*, *COL3A1*, *BMP4*, *SPARC*
Ge et al. (2021)	Mouse and *Cynomolgus* monkey^a^	Young: 3 months, Old: 24 months	Male	Coronary arteries, Aortic arches, Cardiac vasculature	Master regulator of aging ECs	*BACH1*
Xie et al. (2022)	Mouse	4, 26, and 86 weeks	Male	Aorta (Ascending, Arch, Thoracic)	Identified VSMC markers	*Acta2*, *Cnn1*
Liu et al. (2023)	Mouse	6–8 weeks (young), 48 months (old)	Female	Cardiac microvasculature	Proinflammatory phenotype subclusters for ECs	*S100a8/a9*, *CD74*, *VCAM1*
Xie et al. (2022)	Mouse	4, 26, and 86 weeks	Male	Aorta (Ascending, Arch, Thoracic)	EC1: Senescence, Inflammation, EC2: Vascular Tone, NO production	*EC1*: *Cd36*, *Rgcc*, *Gpihbp1*; *EC2*: *Plvap*, *Vwf*
Kalluri et al. (2019)	Mouse	12 weeks; ±8 weeks Western diet	Female	Whole aorta (root to femoral bifurcation; ascending, arch, descending thoracic, abdominal)	EC1: ECM organization/integrin interaction ECs; EC2: lipoprotein‐handling/angiogenic ECs; EC3: lymphatic ECs	EC1: Vcam1, Clu, Gkn3, Eln; EC2: Cd36, Fabp4, Lpl, Gpihbp1, Flt1; EC3: Lyve1
Terekhova et al. (2023)	Human	25–85 years	Male and Female	PBMC (peripheral blood)	Type 2 memory CD4+ T cells; CCR4+ CD8+ Tcm (type 2‐like); HLA‐DR+ CD4+ memory T cells; GZMK+ CD8+ T cells; NKG2C+ GZMB− CD8+ Tmem	Type 2: IL4, CCR4, GATA3; CD4+ HLA‐DR+ Tmem: HLA‐DR; CD8+ Tem: GZMK; NKG2C+ subset: KLRC2 (NKG2C), XCL1

^a^ *Macaca fascicularis*.

**TABLE 2:** Summary of single‐cell transcriptomic studies on cellular heterogeneity in arterial aging with additional risk factors.

Study	Species	Ages	Gender	Artery/Microvascular site	Additional risk factors	Subsets	Markers
Cheng et al. (2024)	Rat	16–18 weeks	Male	Aortic Artery, Mesenteric Artery	Hypertension	Metabolic regulation SMC; ECM remodeling SMC; ECM‐associated SMC; Stress‐responsive SMC; Pro‐inflammatory SMC	*Hmgcs2*, *Bcr*; *Fbln5*; *Lum*; *Hspb1*, *Hspa1b*; *Nfkbia*, *Nfkb2*, *Il6*
Smit et al. (2023)	Mouse	5 months (young), 22 months (old)	Female	Aortic arch (atherosclerotic plaques)	Hypercholesterolemia (Ldlr^−/−^ HCD)	Age‐associated GzmK^+^ CD8^+^ T cells; Age‐associated B cells (ABCs)	Gzmk, Prf1, Pdcd1, Lag3, Nkg7, Ccl5; Cd11b (Itgam), Cd11c (Itgax), Tbx21, H2‐Ab1, Cd80, Cd86
Cheng et al. (2024)	Rat	16 weeks (young), 72 weeks (old)	Male	Aorta, Femoral artery, Mesentery artery	Hypertension (Wistar Kyoto, SHR)	Spp1+ synthetic SMCs; Spp1high matrix activated fibroblasts; Spp1high scar‐associated macrophage	*Spp1*
Hartman et al. (2021)	Mouse, Human	Mouse: 16–26 weeks, Human: 69 years	Male & Female	Brachiocephalic artery, Carotid endarterectomy	Advanced atherosclerotic lesions (ApoE−/−)	Phenotypically modulated SMCs Female‐enriched in SMCs	*Fn1, Mfap4* *GAS6*, SERPING1

## Author Contributions

J.S. conceived the original idea for the manuscript, proofread, and commented on final as well as intermediate manuscript versions. H.X. drafted the initial manuscript, designed the graphical abstract, and collected data in tables. P.‐L.M. and D.K. rewrote and edited the initial manuscript. D.K. designed and prepared the graphical abstract.

## Funding

This research was funded by the Netherlands Organization for Scientific Research Research (VICI 09150182410051); and a Humboldt fellowship to J.S., a fellowship from the Chinese scholarship Council to H.X., and a CARIM postdoctoral fellowship to P.‐L.M.

## Conflicts of Interest

The authors declare no conflicts of interest.

## Data Availability

Data sharing not applicable to this article as no datasets were generated or analysed during the current study.

## References

[acel70597-bib-0001] Ahn, M. , J. Kim , and J. H. Seo . 2025. “Single‐Cell RNA‐Seq Analysis Reveals the Multi‐Step Process of Cellular Senescence.” Biochem Biophys Rep 42: 102042. 10.1016/j.bbrep.2025.102042. 40476058 PMC12138942

[acel70597-bib-0002] Ajoolabady, A. , D. Pratico , M. Vinciguerra , G. Y. H. Lip , C. Franceschi , and J. Ren . 2023. “Inflammaging: Mechanisms and Role in the Cardiac and Vasculature.” Trends in Endocrinology and Metabolism 34, no. 6: 373–387. 10.1016/j.tem.2023.03.005. 37076375

[acel70597-bib-0003] Akhiyat, N. , E. Hellou , I. Ozcan , et al. 2025. “Endothelial Dysfunction as a Feature of Vascular Aging.” European Journal of Preventive Cardiology 10: zwaf544. 10.1093/eurjpc/zwaf544. 41069066

[acel70597-bib-0004] Aranda, J. F. , C. M. Ramírez , and M. Mittelbrunn . 2025. “Inflammageing, a Targetable Pathway for Preventing Cardiovascular Diseases.” Cardiovascular Research 121, no. 10: 1537–1550. 10.1093/cvr/cvae240. 39530590 PMC12391671

[acel70597-bib-0005] Avelar, R. A. , J. G. Ortega , R. Tacutu , et al. 2020. “A Multidimensional Systems Biology Analysis of Cellular Senescence in Aging and Disease.” Genome Biology 21, no. 1: 91. 10.1186/s13059-020-01990-9. 32264951 PMC7333371

[acel70597-bib-0006] Bakhshian Nik, A. , H. H. Ng , S. K. Ashbrook , et al. 2023. “Epidermal Growth Factor Receptor Inhibition Prevents Vascular Calcifying Extracellular Vesicle Biogenesis.” American Journal of Physiology. Heart and Circulatory Physiology 324, no. 4: H553–H570. 10.1152/ajpheart.00280.2022. 36827229 PMC10042607

[acel70597-bib-0007] Barcena, M. L. , M. Aslam , S. Pozdniakova , K. Norman , and Y. Ladilov . 2022. “Cardiovascular Inflammaging: Mechanisms and Translational Aspects.” Cells 11, no. 6: 1010. https://www.mdpi.com/2073‐4409/11/6/1010. 35326461 10.3390/cells11061010 PMC8946971

[acel70597-bib-0008] Beenakker, J. W. , B. A. Ashcroft , J. H. Lindeman , and T. H. Oosterkamp . 2012. “Mechanical Properties of the Extracellular Matrix of the Aorta Studied by Enzymatic Treatments.” Biophysical Journal 102, no. 8: 1731–1737. 10.1016/j.bpj.2012.03.041. 22768928 PMC3328707

[acel70597-bib-0009] Bloom, S. I. , M. T. Islam , L. A. Lesniewski , and A. J. Donato . 2023. “Mechanisms and Consequences of Endothelial Cell Senescence.” Nature Reviews Cardiology 20, no. 1: 38–51. 10.1038/s41569-022-00739-0. 35853997 PMC10026597

[acel70597-bib-0010] Boulanger, C. M. 2018. “Highlight on Endothelial Activation and Beyond.” Arteriosclerosis, Thrombosis, and Vascular Biology 38, no. 12: e198–e201. 10.1161/ATVBAHA.118.312054. 30571176

[acel70597-bib-0011] Boyer, M. J. , Y. Kimura , T. Akiyama , et al. 2020. “Endothelial Cell‐Derived Extracellular Vesicles Alter Vascular Smooth Muscle Cell Phenotype Through High‐Mobility Group Box Proteins.” Journal of Extracellular Vesicles 9, no. 1: 1781427. 10.1080/20013078.2020.1781427. 32944170 PMC7480479

[acel70597-bib-0012] Buechler, M. B. , R. N. Pradhan , A. T. Krishnamurty , et al. 2021. “Cross‐Tissue Organization of the Fibroblast Lineage.” Nature 593, no. 7860: 575–579. 10.1038/s41586-021-03549-5. 33981032

[acel70597-bib-0013] Chan, G. H. H. , and R. R. Fiscus . 2004. “Exaggerated Production of Nitric Oxide (NO) and Increases in Inducible NO‐Synthase mRNA Levels Induced by the Pro‐Inflammatory Cytokine Interleukin‐1β in Vascular Smooth Muscle Cells of Elderly Rats.” Experimental Gerontology 39, no. 3: 387–394. 10.1016/j.exger.2004.01.002. 15036398

[acel70597-bib-0014] Cheng, J. , H. Wu , C. Xie , et al. 2024. “Single‐Cell Mapping of Large and Small Arteries During Hypertensive Aging.” Journals of Gerontology. Series A, Biological Sciences and Medical Sciences 79, no. 2: glad188. 10.1093/gerona/glad188. 37531301

[acel70597-bib-0015] Ciceri, P. , F. Elli , L. Cappelletti , et al. 2016. “Osteonectin (SPARC) Expression in Vascular Calcification: In Vitro and Ex Vivo Studies.” Calcified Tissue International 99, no. 5: 472–480. 10.1007/s00223-016-0167-x. 27339669

[acel70597-bib-0016] Cui, H. , C. Wang , H. Maan , et al. 2024. “scGPT: Toward Building a Foundation Model for Single‐Cell Multi‐Omics Using Generative AI.” Nature Methods 21, no. 8: 1470–1480. 10.1038/s41592-024-02201-0. 38409223

[acel70597-bib-0017] Dai, D. , S. Gu , X. Han , et al. 2024. “The Transcription Factor ZEB2 Drives the Formation of Age‐Associated B Cells.” Science 383, no. 6681: 413–421. 10.1126/science.adf8531. 38271512 PMC7616037

[acel70597-bib-0018] Dhore, C. R. , J. P. Cleutjens , E. Lutgens , et al. 2001. “Differential Expression of Bone Matrix Regulatory Proteins in Human Atherosclerotic Plaques.” Arteriosclerosis, Thrombosis, and Vascular Biology 21, no. 12: 1998–2003. 10.1161/hq1201.100229. 11742876

[acel70597-bib-0019] Dobner, S. , L. Kleissl , F. Tóth , et al. 2025. “Uncovering the Transcriptional Hallmarks of Endothelial Cell Aging via Integrated Single‐Cell Analysis.” bioRxiv, 2025.2008.2018.669055. 10.1101/2025.08.18.669055.

[acel70597-bib-0020] Dutzmann, J. , J. M. Daniel , L. Korte , et al. 2025. “Adventitial Fibroblasts Release Interleukin 6 After Vascular Injury and Induce Smooth Muscle Cell Proliferation and Neointima Formation.” Journal of the American Heart Association 14, no. 14: e040143. 10.1161/jaha.124.040143. 40611486 PMC12533623

[acel70597-bib-0021] Elyahu, Y. , I. Hekselman , I. Eizenberg‐Magar , et al. 2019. “Aging Promotes Reorganization of the CD4 T Cell Landscape Toward Extreme Regulatory and Effector Phenotypes.” Science Advances 5, no. 8: eaaw8330. 10.1126/sciadv.aaw8330. 31457092 PMC6703865

[acel70597-bib-0022] Filippov, I. , L. Schauser , and P. Peterson . 2024. “An Integrated Single‐Cell Atlas of Blood Immune Cells in Aging.” NPJ Aging 10, no. 1: 59. 10.1038/s41514-024-00185-x. 39613786 PMC11606963

[acel70597-bib-0023] Fleenor, B. S. , K. D. Marshall , J. R. Durrant , L. A. Lesniewski , and D. R. Seals . 2010. “Arterial Stiffening With Ageing Is Associated With Transforming Growth Factor‐β1‐Related Changes in Adventitial Collagen: Reversal by Aerobic Exercise.” Journal of Physiology 588, no. 20: 3971–3982. 10.1113/jphysiol.2010.194753. 20807791 PMC3000586

[acel70597-bib-0024] Ge, F. , Q. Pan , Y. Qin , et al. 2021. “Single‐Cell Analysis Identify Transcription Factor BACH1 as a Master Regulator Gene in Vascular Cells During Aging.” Frontiers in Cell and Development Biology 9: 786496. 10.3389/fcell.2021.786496. PMC8740196 35004685

[acel70597-bib-0025] Goncalves, I. , M. Pan , P. Singh , et al. 2026. “Spatial Transcriptomics Reveals a Key Role of Fibroblast‐Like Vascular Smooth Muscle Cells in Human Atherosclerotic Cell Crosstalk and Stability.” European Heart Journal 13: ehaf1091. 10.1093/eurheartj/ehaf1091. PMC13384730 41685669

[acel70597-bib-0026] Gong, Q. , M. Sharma , M. C. Glass , et al. 2025. “Multi‐Omic Profiling Reveals Age‐Related Immune Dynamics in Healthy Adults.” Nature 648, no. 8094: 696–706. 10.1038/s41586-025-09686-5. 41162704 PMC12711581

[acel70597-bib-0027] Gorgoulis, V. , P. D. Adams , A. Alimonti , et al. 2019. “Cellular Senescence: Defining a Path Forward.” Cell 179, no. 4: 813–827. 10.1016/j.cell.2019.10.005. 31675495

[acel70597-bib-0028] Grover, A. , A. Sanjuan‐Pla , S. Thongjuea , et al. 2016. “Single‐Cell RNA Sequencing Reveals Molecular and Functional Platelet Bias of Aged Haematopoietic Stem Cells.” Nature Communications 7, no. 1: 11075. 10.1038/ncomms11075. PMC4820843 27009448

[acel70597-bib-0029] Guzik, T. J. , and T. Mikolajczyk . 2014. “In Search of the T Cell Involved in Hypertension and Target Organ Damage.” Hypertension 64, no. 2: 224–226. 10.1161/HYPERTENSIONAHA.114.03340. 24866136

[acel70597-bib-0030] Hamczyk, M. R. , R. M. Nevado , P. Gonzalo , et al. 2024. “Endothelial‐To‐Mesenchymal Transition Contributes to Accelerated Atherosclerosis in Hutchinson‐Gilford Progeria Syndrome.” Circulation 150, no. 20: 1612–1630. 10.1161/CIRCULATIONAHA.123.065768. 39206565

[acel70597-bib-0031] Han, Y. , and S. Y. Kim . 2023. “Endothelial Senescence in Vascular Diseases: Current Understanding and Future Opportunities in Senotherapeutics.” Experimental & Molecular Medicine 55, no. 1: 1–12. 10.1038/s12276-022-00906-w. 36599934 PMC9898542

[acel70597-bib-0032] Harrison, C. B. , S. C. Trevelin , D. A. Richards , et al. 2021. “Fibroblast Nox2 (NADPH Oxidase‐2) Regulates ANG II (Angiotensin II)‐Induced Vascular Remodeling and Hypertension via Paracrine Signaling to Vascular Smooth Muscle Cells.” Arteriosclerosis, Thrombosis, and Vascular Biology 41, no. 2: 698–710. 10.1161/atvbaha.120.315322. 33054395 PMC7837692

[acel70597-bib-0033] Hartman, R. J. G. , K. Owsiany , L. Ma , et al. 2021. “Sex‐Stratified Gene Regulatory Networks Reveal Female Key Driver Genes of Atherosclerosis Involved in Smooth Muscle Cell Phenotype Switching.” Circulation 143, no. 7: 713–726. 10.1161/circulationaha.120.051231. 33499648 PMC7930467

[acel70597-bib-0034] Haschak, M. , S. LoPresti , E. Stahl , S. Dash , B. Popovich , and B. N. Brown . 2021. “Macrophage Phenotype and Function Are Dependent Upon the Composition and Biomechanics of the Local Cardiac Tissue Microenvironment.” Aging (Albany NY) 13, no. 13: 16938–16956. 10.18632/aging.203054. 34292877 PMC8312435

[acel70597-bib-0035] Hayashi, K. , S. Nakamura , W. Nishida , and K. Sobue . 2006. “Bone Morphogenetic Protein‐Induced MSX1 and MSX2 Inhibit Myocardin‐Dependent Smooth Muscle Gene Transcription.” Molecular and Cellular Biology 26, no. 24: 9456–9470. 10.1128/mcb.00759-06. 17030628 PMC1698541

[acel70597-bib-0036] Hays, T. T. , B. Ma , N. Zhou , S. Stoll , W. J. Pearce , and H. Qiu . 2018. “Vascular Smooth Muscle Cells Direct Extracellular Dysregulation in Aortic Stiffening of Hypertensive Rats.” Aging Cell 17, no. 3: e12748. 10.1111/acel.12748. 29603864 PMC5946086

[acel70597-bib-0037] Herzog, M. J. , P. Müller , K. Lechner , et al. 2025. “Arterial Stiffness and Vascular Aging: Mechanisms, Prevention, and Therapy.” Signal Transduction and Targeted Therapy 10, no. 1: 282. 10.1038/s41392-025-02346-0. 40887468 PMC12399776

[acel70597-bib-0038] Hirase, T. , and K. Node . 2012. “Endothelial Dysfunction as a Cellular Mechanism for Vascular Failure.” American Journal of Physiology. Heart and Circulatory Physiology 302, no. 3: H499–H505. 10.1152/ajpheart.00325.2011. 22081698

[acel70597-bib-0039] Hoeft, K. , G. J. L. Schaefer , H. Kim , et al. 2023. “Platelet‐Instructed SPP1(+) Macrophages Drive Myofibroblast Activation in Fibrosis in a CXCL4‐Dependent Manner.” Cell Reports 42, no. 2: 112131. 10.1016/j.celrep.2023.112131. 36807143 PMC9992450

[acel70597-bib-0040] Hoffmann, G. , S. Kenn , B. Wirleitner , et al. 1998. “Neopterin Induces Nitric Oxide‐Dependent Apoptosis in Rat Vascular Smooth Muscle Cells.” Immunobiology 199, no. 1: 63–73. 10.1016/S0171-2985(98)80064-8. 9717668

[acel70597-bib-0041] Hu, Z. , W. Liu , X. Hua , et al. 2021. “Single‐Cell Transcriptomic Atlas of Different Human Cardiac Arteries Identifies Cell Types Associated With Vascular Physiology.” Arteriosclerosis, Thrombosis, and Vascular Biology 41, no. 4: 1408–1427. 10.1161/ATVBAHA.120.315373. 33626908

[acel70597-bib-0042] Indranil, B. , and A. K. Gausal . 2019. “Endothelial Dysfunction in Cardiovascular Diseases.” In Basic and Clinical Understanding of Microcirculation (Pp. Ch. 5), edited by S. K. Fatima , S. S. S. Saeedi , and B. N. Luqman . IntechOpen. 10.5772/intechopen.89365.

[acel70597-bib-0043] Kalluri, A. S. , S. K. Vellarikkal , E. R. Edelman , et al. 2019. “Single‐Cell Analysis of the Normal Mouse Aorta Reveals Functionally Distinct Endothelial Cell Populations.” Circulation 140, no. 2: 147–163. 10.1161/circulationaha.118.038362. 31146585 PMC6693656

[acel70597-bib-0044] Khan, S. , M. Chakraborty , F. Wu , et al. 2026. “B Cells Drive CD4 T Cell Immunosenescence and Age‐Associated Health Decline.” Science Immunology 11, no. 115: eadv7615. 10.1126/sciimmunol.adv7615. 41616067 PMC13137872

[acel70597-bib-0045] Kielty, C. M. , S. Stephan , M. J. Sherratt , M. Williamson , and C. A. Shuttleworth . 2007. “Applying Elastic Fibre Biology in Vascular Tissue Engineering.” Philosophical Transactions of the Royal Society of London. Series B, Biological Sciences 362, no. 1484: 1293–1312. 10.1098/rstb.2007.2134. 17588872 PMC2440413

[acel70597-bib-0046] Kitada, M. , Y. Ogura , and D. Koya . 2016. “The Protective Role of Sirt1 in Vascular Tissue: Its Relationship to Vascular Aging and Atherosclerosis.” Aging 8, no. 10: 2290–2307. 10.18632/aging.101068. 27744418 PMC5115889

[acel70597-bib-0047] Kleshchevnikov, V. , A. Shmatko , E. Dann , et al. 2022. “Cell2location Maps Fine‐Grained Cell Types in Spatial Transcriptomics.” Nature Biotechnology 40, no. 5: 661–671. 10.1038/s41587-021-01139-4. 35027729

[acel70597-bib-0048] Kovacic, J. C. , P. Moreno , E. G. Nabel , V. Hachinski , and V. Fuster . 2011. “Cellular Senescence, Vascular Disease, and Aging.” Circulation 123, no. 17: 1900–1910. 10.1161/CIRCULATIONAHA.110.009118. 21537006

[acel70597-bib-0049] Krajnik, A. , E. Nimmer , J. A. Brazzo 3rd , et al. 2023. “Survivin Regulates Intracellular Stiffness and Extracellular Matrix Production in Vascular Smooth Muscle Cells.” APL Bioengineering 7, no. 4: 46104. 10.1063/5.0157549. PMC10590228 37868708

[acel70597-bib-0050] Kroemer, G. , A. B. Maier , A. M. Cuervo , et al. 2025. “From Geroscience to Precision Geromedicine: Understanding and Managing Aging.” Cell 188, no. 8: 2043–2062. 10.1016/j.cell.2025.03.011. 40250404 PMC12037106

[acel70597-bib-0051] Lacolley, P. , V. Regnault , P. Segers , and S. Laurent . 2017. “Vascular Smooth Muscle Cells and Arterial Stiffening: Relevance in Development, Aging, and Disease.” Physiological Reviews 97, no. 4: 1555–1617. 10.1152/physrev.00003.2017. 28954852

[acel70597-bib-0052] Lagger, C. , E. Ursu , A. Equey , et al. 2021. “scAgeCom: A Murine Atlas of Age‐Related Changes in Intercellular Communication Inferred With the Package scDiffCom.” bioRxiv, 2021.2008.2013.456238. 10.1101/2021.08.13.456238.

[acel70597-bib-0053] Lagger, C. , E. Ursu , A. Equey , et al. 2023. “scDiffCom: A Tool for Differential Analysis of Cell–Cell Interactions Provides a Mouse Atlas of Aging Changes in Intercellular Communication.” Nature Aging 3, no. 11: 1446–1461. 10.1038/s43587-023-00514-x. 37919434 PMC10645595

[acel70597-bib-0054] Lai, A. , Y. Zhou , C. Chheang , et al. 2026. “Decoding Vascular Aging: Substrate Stiffness and Shear Stress Orchestrate Endothelial Inflammation and Remodelling via Mechanosensitive Pathways.” Biomaterials 329: 123932. 10.1016/j.biomaterials.2025.123932. 41435451

[acel70597-bib-0055] Lanzer, P. , F. M. Hannan , J. D. Lanzer , et al. 2021. “Medial Arterial Calcification: JACC State‐Of‐The‐Art Review.” Journal of the American College of Cardiology 78, no. 11: 1145–1165. 10.1016/j.jacc.2021.06.049. 34503684 PMC8439554

[acel70597-bib-0056] Lanzer, P. , L. Schurgers , A. Twarda‐Clapa , et al. 2025. “Medial Arterial Calcification in Ageing and Disease: Current Evidence and Knowledge Gaps.” European Heart Journal 46, no. 45: 4876–4900. 10.1093/eurheartj/ehaf341. 40574608 PMC13376153

[acel70597-bib-0057] Lebas, M. , G. Chinigò , E. Courmont , et al. 2024. “Integrated Single‐Cell RNA‐Seq Analysis Reveals Mitochondrial Calcium Signaling as a Modulator of Endothelial‐To‐Mesenchymal Transition.” Science Advances 10, no. 32: eadp6182. 10.1126/sciadv.adp6182. 39121218 PMC11313856

[acel70597-bib-0058] Lee, H.‐Y. , and B.‐H. Oh . 2010. “Aging and Arterial Stiffness.” Circulation Journal 74, no. 11: 2257–2262. 10.1253/circj.CJ-10-0910. 20962429

[acel70597-bib-0059] Liu, Y. , M. Zuo , A. Wu , et al. 2025. “UFMylation Maintains YAP Stability to Promote Vascular Endothelial Cell Senescence.” iScience 28, no. 2: 111854. 10.1016/j.isci.2025.111854. 39991547 PMC11847039

[acel70597-bib-0060] Liu, Z. , Y. Huang , D. Wang , et al. 2023. “Insights Gained From Single‐Cell RNA Analysis of Murine Endothelial Cells in Aging Hearts.” Heliyon 9, no. 8: e18324. 10.1016/j.heliyon.2023.e18324. 37554834 PMC10404962

[acel70597-bib-0061] Longtine, A. G. , N. T. Greenberg , Y. Bernaldo de Quirós , and V. E. Brunt . 2024. “The Gut Microbiome as a Modulator of Arterial Function and Age‐Related Arterial Dysfunction.” American Journal of Physiology. Heart and Circulatory Physiology 326, no. 4: H986–H1005. 10.1152/ajpheart.00764.2023. 38363212 PMC11279790

[acel70597-bib-0062] López‐Otín, C. , M. A. Blasco , L. Partridge , M. Serrano , and G. Kroemer . 2023. “Hallmarks of Aging: An Expanding Universe.” Cell 186, no. 2: 243–278. 10.1016/j.cell.2022.11.001. 36599349

[acel70597-bib-0063] Luu, N. , A. Bajpai , R. Li , et al. 2024. “Aging‐Associated Decline in Vascular Smooth Muscle Cell Mechanosensation Is Mediated by Piezo1 Channel.” Aging Cell 23, no. 2: e14036. 10.1111/acel.14036. 37941511 PMC10861209

[acel70597-bib-0064] Ma, S. , and V. N. Gladyshev . 2017. “Molecular Signatures of Longevity: Insights From Cross‐Species Comparative Studies.” Seminars in Cell & Developmental Biology 70: 190–203. 10.1016/j.semcdb.2017.08.007. 28800931 PMC5807068

[acel70597-bib-0065] Majewska, J. , and V. Krizhanovsky . 2025. “Immune Surveillance of Senescent Cells in Aging and Disease.” Nature Aging 5, no. 8: 1415–1424. 10.1038/s43587-025-00910-5. 40813810

[acel70597-bib-0066] Martinez‐Jimenez, C. P. , N. Eling , H.‐C. Chen , et al. 2017. “Aging Increases Cell‐To‐Cell Transcriptional Variability Upon Immune Stimulation.” Science 355, no. 6332: 1433–1436. 10.1126/science.aah4115. 28360329 PMC5405862

[acel70597-bib-0067] Mazan‐Mamczarz, K. , D. Tsitsipatis , B. G. Childs , et al. 2025. “Single‐Cell and Spatial Transcriptomics Map Senescent Vascular Cells in Arterial Remodeling During Atherosclerosis in Mice.” Nature Aging 5, no. 8: 1528–1547. 10.1038/s43587-025-00889-z. 40660002 PMC12323397

[acel70597-bib-0068] Méndez‐Barbero, N. , C. Gutiérrez‐Muñoz , and L. M. Blanco‐Colio . 2021. “Cellular Crosstalk Between Endothelial and Smooth Muscle Cells in Vascular Wall Remodeling.” International Journal of Molecular Sciences 22, no. 14: 7284. 10.3390/ijms22147284. 34298897 PMC8306829

[acel70597-bib-0069] Michel, J.‐B. , J. Lagrange , V. Regnault , and P. Lacolley . 2022. “Conductance Artery Wall Layers and Their Respective Roles in the Clearance Functions.” Arteriosclerosis, Thrombosis, and Vascular Biology 42, no. 9: e253–e272. 10.1161/ATVBAHA.122.317759. 35924557

[acel70597-bib-0070] Mikhaylova, L. , J. Malmquist , and M. Nurminskaya . 2007. “Regulation of In Vitro Vascular Calcification by BMP4, VEGF and Wnt3a.” Calcified Tissue International 81, no. 5: 372–381. 10.1007/s00223-007-9073-6. 17982705

[acel70597-bib-0071] Najjar, S. S. , A. Scuteri , and E. G. Lakatta . 2005. “Arterial Aging.” Hypertension 46, no. 3: 454–462. 10.1161/01.HYP.0000177474.06749.98. 16103272

[acel70597-bib-0072] Nevalainen, T. , L. Kananen , S. Marttila , et al. 2015. “Transcriptomic and Epigenetic Analyses Reveal a Gender Difference in Aging‐Associated Inflammation: The Vitality 90+ Study.” Age (Dordrecht, Netherlands) 37, no. 4: 9814. 10.1007/s11357-015-9814-9. 26188803 PMC4506741

[acel70597-bib-0073] Osherov, A. B. , L. Gotha , A. N. Cheema , B. Qiang , and B. H. Strauss . 2011. “Proteins Mediating Collagen Biosynthesis and Accumulation in Arterial Repair: Novel Targets for Anti‐Restenosis Therapy.” Cardiovascular Research 91, no. 1: 16–26. 10.1093/cvr/cvr012. 21245059

[acel70597-bib-0074] Pan, X.‐X. , F. Wu , X.‐H. Chen , et al. 2020. “T‐Cell Senescence Accelerates Angiotensin II‐Induced Target Organ Damage.” Cardiovascular Research 117, no. 1: 271–283. 10.1093/cvr/cvaa032. 32049355

[acel70597-bib-0075] Pauli, J. , D. Garger , F. Peymani , et al. 2025. “Single Cell Spatial Transcriptomics Integration Deciphers the Morphological Heterogeneity of Atherosclerotic Carotid Arteries.” Nature Communications 16, no. 1: 11282. 10.1038/s41467-025-67679-4. PMC12717225 41413386

[acel70597-bib-0076] Piccolo, S. , S. Dupont , and M. Cordenonsi . 2014. “The Biology of YAP/TAZ: Hippo Signaling and Beyond.” Physiological Reviews 94, no. 4: 1287–1312. 10.1152/physrev.00005.2014. 25287865

[acel70597-bib-0077] Qi, D. , M. Wei , S. Jiao , et al. 2019. “Hypoxia Inducible Factor 1α in Vascular Smooth Muscle Cells Promotes Angiotensin II‐Induced Vascular Remodeling via Activation of CCL7‐Mediated Macrophage Recruitment.” Cell Death & Disease 10, no. 8: 544. 10.1038/s41419-019-1757-0. 31320613 PMC6639417

[acel70597-bib-0078] Reed, E. , A. Fellows , R. Lu , et al. 2022. “Extracellular Matrix Profiling and Disease Modelling in Engineered Vascular Smooth Muscle Cell Tissues.” Matrix Biology Plus 16: 100122. 10.1016/j.mbplus.2022.100122. 36193159 PMC9526190

[acel70597-bib-0079] Rivera, C. F. , Y. M. Farra , M. Silvestro , et al. 2024. “Mapping the Unicellular Transcriptome of the Ascending Thoracic Aorta to Changes in Mechanosensing and Mechanoadaptation During Aging.” Aging Cell 23, no. 8: e14197. 10.1111/acel.14197. 38825882 PMC11320362

[acel70597-bib-0080] Saul, D. , R. L. Kosinsky , E. J. Atkinson , et al. 2022. “A New Gene Set Identifies Senescent Cells and Predicts Senescence‐Associated Pathways Across Tissues.” Nature Communications 13, no. 1: 4827. 10.1038/s41467-022-32552-1. PMC9381717 35974106

[acel70597-bib-0081] Saz‐Lara, A. , I. Cavero‐Redondo , C. Pascual‐Morena , et al. 2023. “Early Vascular Aging as an Index of Cardiovascular Risk in Healthy Adults: Confirmatory Factor Analysis From the EVasCu Study.” Cardiovascular Diabetology 22, no. 1: 209. 10.1186/s12933-023-01947-9. 37592251 PMC10436435

[acel70597-bib-0082] Shehadeh, L. A. , K. A. Webster , J. M. Hare , and R. I. Vazquez‐Padron . 2011. “Dynamic Regulation of Vascular Myosin Light Chain (MYL9) With Injury and Aging.” PLoS One 6, no. 10: e25855. 10.1371/journal.pone.0025855. 22003410 PMC3189218

[acel70597-bib-0083] Sladitschek‐Martens, H. L. , A. Guarnieri , G. Brumana , et al. 2022. “YAP/TAZ Activity in Stromal Cells Prevents Ageing by Controlling cGAS‐STING.” Nature 607, no. 7920: 790–798. 10.1038/s41586-022-04924-6. 35768505 PMC7613988

[acel70597-bib-0084] Slenders, L. , M. Wesseling , S. Wei , et al. 2025. “Endothelial‐To‐Mesenchymal Transition Gene Signature Derived From Single‐Cell Transcriptomics of Human Atherosclerotic Tissue Associates With Stable Plaque Histological Characteristics.” Vascular Pharmacology 159: 107498. 10.1016/j.vph.2025.107498. 40318741

[acel70597-bib-0085] Smit, V. , J. de Mol , F. H. Schaftenaar , et al. 2023. “Single‐Cell Profiling Reveals Age‐Associated Immunity in Atherosclerosis.” Cardiovascular Research 119, no. 15: 2508–2521. 10.1093/cvr/cvad099. 37390467 PMC10676459

[acel70597-bib-0086] Souilhol, C. , M. C. Harmsen , P. C. Evans , and G. Krenning . 2018. “Endothelial–Mesenchymal Transition in Atherosclerosis.” Cardiovascular Research 114, no. 4: 565–577. 10.1093/cvr/cvx253. 29309526

[acel70597-bib-0087] Spray, L. , G. Richardson , L. K. Booth , et al. 2025. “How to Measure and Model Cardiovascular Aging.” Cardiovascular Research 121, no. 10: 1489–1508. 10.1093/cvr/cvaf138. 40873304 PMC12391676

[acel70597-bib-0088] Stenmark, K. R. , M. E. Yeager , K. C. El Kasmi , et al. 2013. “The Adventitia: Essential Regulator of Vascular Wall Structure and Function.” Annual Review of Physiology 75: 23–47. 10.1146/annurev-physiol-030212-183802. PMC3762248 23216413

[acel70597-bib-0089] Sun, F. , X. Chen , S. Zhang , et al. 2024. “Cross‐Species Signaling Pathways Analysis Inspire Animal Model Selections for Drug Screening and Target Prediction in Vascular Aging Diseases.” Evolutionary Applications 17, no. 6: e13708. 10.1111/eva.13708. 38863828 PMC11164676

[acel70597-bib-0090] Tao, W. , Z. Yu , and J. J. Han . 2024. “Single‐Cell Senescence Identification Reveals Senescence Heterogeneity, Trajectory, and Modulators.” Cell Metabolism 36, no. 5: 1126–1143. 10.1016/j.cmet.2024.03.009. 38604170

[acel70597-bib-0091] Terekhova, M. , A. Swain , P. Bohacova , et al. 2023. “Single‐Cell Atlas of Healthy Human Blood Unveils Age‐Related Loss of NKG2C+GZMB−CD8+ Memory T Cells and Accumulation of Type 2 Memory T Cells.” Immunity 56, no. 12: 2836–2854. 10.1016/j.immuni.2023.10.013. 37963457

[acel70597-bib-0092] Tinajero, M. G. , and A. I. Gotlieb . 2020. “Recent Developments in Vascular Adventitial Pathobiology: The Dynamic Adventitia as a Complex Regulator of Vascular Disease.” American Journal of Pathology 190, no. 3: 520–534. 10.1016/j.ajpath.2019.10.021. 31866347

[acel70597-bib-0093] Tyshkovskiy, A. , S. Ma , A. V. Shindyapina , et al. 2023. “Distinct Longevity Mechanisms Across and Within Species and Their Association With Aging.” Cell 186, no. 13: 2929–2949. 10.1016/j.cell.2023.05.002. 37269831 PMC11192172

[acel70597-bib-0094] Ungvari, Z. , S. Tarantini , A. J. Donato , V. Galvan , and A. Csiszar . 2018. “Mechanisms of Vascular Aging.” Circulation Research 123, no. 7: 849–867. 10.1161/circresaha.118.311378. 30355080 PMC6248882

[acel70597-bib-0095] van Beek, A. A. , J. Van den Bossche , P. G. Mastroberardino , M. P. J. de Winther , and P. J. M. Leenen . 2019. “Metabolic Alterations in Aging Macrophages: Ingredients for Inflammaging?” Trends in Immunology 40, no. 2: 113–127. 10.1016/j.it.2018.12.007. 30626541

[acel70597-bib-0096] van der Linden, J. , S. J. M. Stefens , J. M. Heredia‐Genestar , et al. 2024. “Ercc1 DNA Repair Deficiency Results in Vascular Aging Characterized by VSMC Phenotype Switching, ECM Remodeling, and an Increased Stress Response.” Aging Cell 23, no. 5: e14126. 10.1111/acel.14126. 38451018 PMC11113264

[acel70597-bib-0097] van Kuijk, K. , I. R. McCracken , R. J. H. A. Tillie , et al. 2023. “Human and Murine Fibroblast Single‐Cell Transcriptomics Reveals Fibroblast Clusters Are Differentially Affected by Ageing and Serum Cholesterol.” Cardiovascular Research 119, no. 7: 1509–1523. 10.1093/cvr/cvad016. 36718802 PMC10318398

[acel70597-bib-0098] Verhamme, P. , and M. F. Hoylaerts . 2006. “The Pivotal Role of the Endothelium in Haemostasis and Thrombosis.” Acta Clinica Belgica 61, no. 5: 213–219. 10.1179/acb.2006.036. 17240734

[acel70597-bib-0099] Weinsaft, J. W. , and J. M. Edelberg . 2001. “Aging‐Associated Changes in Vascular Activity: A Potential Link to Geriatric Cardiovascular Disease.” American Journal of Geriatric Cardiology 10, no. 6: 348–354. 10.1111/j.1076-7460.2001.00833.x. 11684920

[acel70597-bib-0100] Wells, S. B. , D. B. Rainbow , M. Mark , et al. 2025. “Multimodal Profiling Reveals Tissue‐Directed Signatures of Human Immune Cells Altered With Age.” Nature Immunology 26, no. 9: 1612–1625. 10.1038/s41590-025-02241-4. 40804529 PMC12396968

[acel70597-bib-0101] WHO . 2021. “Decade of Healthy Ageing: Baseline Report.” W. H. Organization. https://apps.who.int/iris/handle/10665/338677.

[acel70597-bib-0102] Xie, W. , Y. Ke , Q. You , et al. 2022. “Single‐Cell RNA Sequencing and Assay for Transposase‐Accessible Chromatin Using Sequencing Reveals Cellular and Molecular Dynamics of Aortic Aging in Mice.” Arteriosclerosis, Thrombosis, and Vascular Biology 42, no. 2: 156–171. 10.1161/atvbaha.121.316883. 34879708

[acel70597-bib-0103] Yin, Y. , C. Huang , Z. Wang , P. Huang , and S. Qin . 2022. “Identification of Cellular Heterogeneity and Key Signaling Pathways Associated With Vascular Remodeling and Calcification in Young and Old Primate Aortas Based on Single‐Cell Analysis.” Aging (Albany NY) 15, no. 4: 982–1003. 10.18632/aging.204442. 36566020 PMC10008505

[acel70597-bib-0104] Zalewski, A. , and Y. Shi . 1997. “Vascular Myofibroblasts.” Arteriosclerosis, Thrombosis, and Vascular Biology 17, no. 3: 417–422. 10.1161/01.ATV.17.3.417. 9102158

[acel70597-bib-0105] Zernecke, A. , F. Erhard , T. Weinberger , et al. 2023. “Integrated Single‐Cell Analysis‐Based Classification of Vascular Mononuclear Phagocytes in Mouse and Human Atherosclerosis.” Cardiovascular Research 119, no. 8: 1676–1689. 10.1093/cvr/cvac161. 36190844 PMC10325698

[acel70597-bib-0106] Zha, Y. , W. Zhuang , Y. Yang , Y. Zhou , H. Li , and J. Liang . 2022. “Senescence in Vascular Smooth Muscle Cells and Atherosclerosis.” Frontiers in Cardiovascular Medicine 9: 910580. 10.3389/fcvm.2022.910580. 35722104 PMC9198250

[acel70597-bib-0107] Zhang, W. , S. Zhang , P. Yan , et al. 2020. “A Single‐Cell Transcriptomic Landscape of Primate Arterial Aging.” Nature Communications 11, no. 1: 2202. 10.1038/s41467-020-15997-0. PMC7200799 32371953

